# Analysis of Polycyclic Aromatic Hydrocarbons via GC-MS/MS and Heterocyclic Amines via UPLC-MS/MS in Crispy Pork Spareribs for Studying Their Formation during Frying

**DOI:** 10.3390/foods13020185

**Published:** 2024-01-05

**Authors:** Yu-Wen Lai, Baskaran Stephen Inbaraj, Bing-Huei Chen

**Affiliations:** 1 Department of Food Science, Fu Jen Catholic University, New Taipei City 242062, Taiwan; linda871123@gmail.com (Y.-W.L.); sinbaraj@yahoo.com (B.S.I.); 2 Department of Nutrition, China Medical University, Taichung 404328, Taiwan

**Keywords:** heterocyclic amines (HAs), polycyclic aromatic hydrocarbons (PAHs), crispy pork spareribs, QuEChERS, UPLC-MS/MS, GC-MS/MS, frying

## Abstract

This study aims to explore the effects of frying conditions on the formation of HAs and PAHs in crispy pork spareribs, a popular meat commodity sold on Taiwan’s market. Raw pork spareribs were marinated, coated with sweet potato powder, and fried in soybean oil and palm oil at 190 °C/6 min or 150 °C/12 min, followed by an analysis of HAs and PAHs via QuEChERS coupled with UPLC-MS/MS and GC-MS/MS, respectively. Both HAs and PAHs in pork spareribs during frying followed a temperature- and time-dependent rise. A total of 7 HAs (20.34–25.97 μg/kg) and 12 PAHs (67.69–85.10 μg/kg) were detected in pork spareribs fried in soybean oil and palm oil at 150 °C/12 min or 190 °C/6 min, with palm oil producing a higher level of total HAs and a lower level of total PAHs than soybean oil. The content changes of amino acid, reducing sugar, and creatinine played a vital role in affecting HA formation, while the degree of oil unsaturation and the contents of precursors including benzaldehyde, 2-cyclohexene-1-one, and trans,trans-2,4-decadienal showed a crucial role in affecting PAH formation. The principal component analysis revealed that HAs and PAHs were formed by different mechanisms, with the latter being more liable to formation in pork spareribs during frying, while the two-factorial analysis indicated that the interaction between oil type and frying condition was insignificant for HAs and PAHs generated in crispy pork spareribs. Both CcdP (22.67–32.78 μg/kg) and Pyr (16.70–22.36 μg/kg) dominated in PAH formation, while Harman (14.46–17.91 μg/kg) and Norharman (3.41–4.55 μg/kg) dominated in HA formation in crispy pork spareribs during frying. The outcome of this study forms a basis for learning both the variety and content of HAs and PAHs generated during the frying of pork spareribs and the optimum frying condition to minimize their formation.

## 1. Introduction

Many types of toxic compounds such as polycyclic aromatic hydrocarbons (PAHs) and heterocyclic amines (HAs) can be produced during food processing, especially protein-rich meat products. According to a report by the International Agency for Research on Cancer (IARC) [1], processed meat products are classified as a group 1 carcinogen (carcinogenic to humans), while red meat is classified as a group 2A carcinogen (probably carcinogenic to humans). As PAHs and HAs may be carcinogenic to humans or probably carcinogenic to humans, it is imperative to study their formation and inhibition during meat processing.

Structurally, PAHs are composed of two or more aromatic rings with a nonpolar nature and can be generated during the high-temperature roasting or frying of meat products through the incomplete combustion or pyrolysis of organic materials [2]. Based on a report published in 2010 by IARC [3], among the various PAHs, benzo[a]pyrene (BaP) was classified as a group 1 carcinogen, while dibenzo[a,l]pyrene (DBalP), cyclopenta[c,d]pyrene (CcdP), and dibenzo[a,h]anthracene (DBahA) were classified as group 2A carcinogens, as well as dibenzo[a,h]pyrene (DBahP), dibenzo[a,i]pyrene (DBaiP), benzo[j]fluoranthene (BjF), benzo[a]anthracene (BaA), benzo[k]fluoranthene (BkFL), benzo[b]fluoranthene (BbFL), indeno[1,2,3-c,d]pyrene (IP), chrysene (CHR), naphthalene (NaP), and 5-methylchrysene (MCH), which were classified as group 2B carcinogens (possibly carcinogenic to humans). The other PAHs including benzo[g,h,i]perylene (BghiP), dibenzo[a,e]pyrene (DBaeP), pyrene (Pyr), fluoranthene (FL), benzo[c]fluorene (BcF), anthracene (Ant), fluorene (Flu), phenanthrene (Phe), and acenaphthene (AcP) were classified as group 3 (not classified as human carcinogens).

HAs are composed of more than one heterocyclic ring and aromatic ring with/without an amino group and can be produced during the processing of meat products at high temperatures through the interaction of amino acids, reducing sugar, creatine, and creatinine present in meat products [2]. Based on a report published in 1993 by IARC [4], among the various HAs that may be present in foods/food products, 2-amino-3-methyl-imidazo[4,5-f]-quinoline (IQ) was classified as group 2A, while 3-amino-1-methyl-5H-pyrido[4,3-b]indole (Trp-P-2), 2-aminodipyrido-[1,2-a:3′,2′-d]imidazole (Glu-P-2), 3-amino-1,4-dimethyl-5H-pyrido[4,3-b]indole (Trp-P-1), 2-amino-6-methyldipyrido-[1,2-a:3′,2′-d] imidazole (Glu-P-1), 2-amino-3-methyl-9H-pyrido[2,3-b]indole (MeAαC), 2-amino-3,4-dimethyl-imidazo[4,5-f]-quinoline (MeIQ), 2-amino-1-methyl-6-phenylimidazo[4,5-b]-pyridine (PhIP), 2-amino-9H-pyrido[2,3-b]indole (AαC), and 2-amino-3,8-dimethyl-imidazo[4,5-f]-quinoxaline (8-MeIQx) were classified as group 2B.

The possible formation and minimization of PAHs or HAs in meat products during processing have been extensively studied and reviewed [5,6,7,8]. For example, Lai et al. [9] studied the formation and inhibition of PAHs and HAs in ground pork during marinating and reported that both PAH and HA contents followed a time-dependent rise, with HAs being more susceptible to formation than PAHs. In a recent study dealing with the formation of PAHs and HAs in pork jerky as affected by processing temperature and flavoring material, Lai et al. [2] reported that pork jerky roasted at 220 °C generated a higher content of PAHs than at 180 °C but did not affect the HA formation. Interestingly, the addition of sugar was shown to reduce the formation of both PAHs and HAs during roasting, while soy sauce minimized the formation of several PAHs and promoted HA formation [2]. In another study dealing with the effects of oil type and processing condition on the formation of PAHs and HAs in pork fiber, frying in lard or sesame oil was shown to produce a higher content of total HAs at 160 °C for 15 min than at 200 °C for 6 min [10]. But for total PAHs, the highest level was shown for pork fiber fried in lard at 200 °C/6 min and the lowest level in lard at 160 °C/15 min [10]. Furthermore, under the same heating condition, the highly unsaturated sesame oil resulted in a higher content of total PAHs and HAs in pork fiber than the more saturated lard [10]. Consequently, the selection of an appropriate oil and processing condition is imperative to minimize the formation of HAs and PAHs in meat products during processing.

Due to the presence of PAHs and HAs in trace amounts (ppb) in meat/meat products, their extraction and purification have been difficult. To overcome this issue, in recent years, several studies have employed QuEChERS, an abbreviation of quick, easy, cheap, effective, rugged, and safe, for the simultaneous extraction and purification of PAHs and HAs from meat products [2,9]. Principally, both PAHs and HAs can be extracted through the addition of inorganic salt to meat samples for the separation of the organic solvent from water via salting out, followed by purification through the addition of an adsorbent to remove interfering compounds and subsequent adsorbent removal via centrifugation. Following extraction and purification via QuEChERS, the various PAHs and HAs in meat samples were analyzed using GC-MS/MS and UPLC-MS/MS, respectively.

Crispy pork spareribs, a popular meat commodity sold on Taiwan’s market, may generate a high level of PAHs and HAs during high-temperature frying, which can be attributed to the high-fat content. Theoretically, lipid oxidation can proceed fast during frying for the subsequent formation of hydroperoxide and degradation into many compounds such as aldehydes, leading to PAH formation [2]. Additionally, amino acids in pork can undergo Strecker degradation to form aldehydes for subsequent reaction with creatinine and pyridine/pyrazine, leading to the formation of HAs [9]. However, no information is available as to the contents of PAHs and HAs in crispy pork spareribs. The objectives of this study were to analyze and explore the formation of PAHs and HAs in crispy pork spareribs as affected by frying condition through extraction via QuEChERS and subsequent analysis of PAHs by GC-MS/MS and HAs using UPLC-MS/MS. Also, the precursors for the formation of PAHs and HAs in crispy pork spareribs were determined.

## 2. Materials and Methods

### 2.1. Materials

A total of 3 kg of raw pork spareribs (120 pieces) was procured from a local market located in New Taipei City, Taiwan. A total of 21 HA standards, including DMIP, IFP, PhIP, Phe-P-1, MeIQ, 4,8-DiMeIQx, 8-MeIQx, 7,8-DiMeIQx, IQx, IQ, IQ[4,5-b], Iso-IQ, MeAαC, AαC, Trp-P-2, Trp-P-1, Glu-P-2, Glu-P-1, Norharman, Harman, and internal standard 4,7,8-TriMeIQx were procured from Toronto Research Chemicals (Downsview, ON, Canada), while 24 PAH standards including DBaiP, DBahP, CcdP, DBahA, DBaeP, DBalP, BghiP, MCH, IP, CHR, BjF, BaA, BbF, BaP, BcF, Phe, Flu, Pyr, FL, Ant, AcP, NaP, AcPy, and internal standard Triphenylene were from Sigma-Aldrich Co. (St Louis, MO, USA). The full names of HA and PAH standards are shown in Tables S1 and S2, respectively.

An Acquity UPLC BEH C18 column (100 × 2.1 mm ID, particle size 1.7 μm) used for HA separation was from Waters Co. (Milford, MA, USA), while a DB-5 MS capillary column (30 m × 0.25 mm ID, film thickness 0.25 μm) for PAH separation was from Agilent Technologies Co. (Palo Alto, CA, USA). The QuEChERS extraction and purification kits were from Yu-Ho Co (New Taipei City, Taiwan). The HPLC-grade solvents including methanol, acetonitrile, acetone, and hexane were from Merck (Darmstadt, Germany).

### 2.2. Processing of Crispy Pork Spareribs

**Figure 1:**
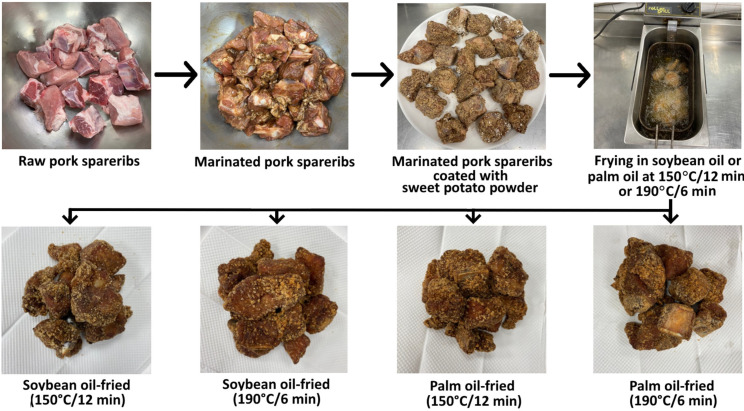
Processing steps of crispy pork spareribs and their appearance after frying in soybean oil or palm oil at different temperatures and time lengths.

The raw pork spareribs (120 pieces) were divided into 3 portions with 1 kg (40 pieces) each. Then, 1 kg of raw pork spareribs from each portion was poured into a saucepan and mixed with a marinade containing 25 g of soy sauce, 25 g of sugar, and 1 g of spice powder containing cinnamon, cumin, coriander, cloves, and star anise. This marinade was selected as it is a standard formula used by many restaurants for the preparation of crispy pork spareribs in Taiwan with a desirable flavor and texture. Furthermore, the incorporation of cinnamon powder (0.5%) was shown to be effective in inhibiting HA and PAH formation in ground pork during marinating [9], while the addition of sugar (20%) was efficient in reducing HA and PAH formation in pork jerky during roasting [2]. After mixing homogeneously, this mixture was stood at 4 °C for 30 min for marinating. Next, each raw pork sparerib was evenly coated with sweet potato powder (3 g) and stood at room temperature for 5 min. Each portion of raw pork spareribs was then poured into a fryer containing 3 L of soybean oil and palm oil separately for frying at 190 °C/6 min and 150 °C/12 min. After frying, the crispy pork spareribs samples were subjected to an analysis of HAs and PAHs via QuEChERS coupled with UPLC-MS/MS and GC-MS/MS, respectively. Triplicate experiments were conducted, and the various HAs and PAHs in pork samples were analyzed in triplicate. The processing step and appearance of products are shown in Figure 1.

### 2.3. Simultaneous Extraction and Purification of HAs and PAHs in Raw Pork Spareribs, Marinated Pork Spareribs, and Crispy Pork Spareribs

A method described by Lai et al. [2,9] was used to extract and purify HAs and PAHs in raw, marinated, and crispy pork spareribs simultaneously. Briefly, 2 g of the pork sample was poured into a centrifuge tube containing one ceramic homogenizer, followed by adding 10 mL of deionized water and shaking this mixture at 200 rpm for 10 min. Then 10 mL of acetonitrile containing 1% acetic acid was added for shaking at 200 rpm for 10 min, after which the extraction powder containing 4 g of magnesium sulfate and 1 g of sodium acetate was added for shaking (1 min), centrifuging at 4 °C for 10 min (4000× *g*), and collecting the supernatant (6 mL) for purification. Next, the supernatant was poured into a centrifuge tube containing 300 mg of PSA, 900 mg of magnesium sulfate, and 300 mg of C18 EC. Following shaking for 1 min and centrifuging at 4 °C for 10 min (4000× *g*), 1 mL of the supernatant was collected, evaporated to dryness under nitrogen, dissolved in 0.2 mL of methanol containing internal standard 4,7,8-TriMeIQx at 1 ppb, and filtered through a 0.22 μm membrane filter for HA analysis via UPLC-MS/MS. For PAH analysis, the residue was dissolved in 0.2 mL of hexane containing internal standard Triphenylene at 10 ppb and filtered through a 0.22 μm membrane filter for PAH analysis via GC-MS/MS.

### 2.4. Analysis of HAs in Raw Pork Spareribs, Marinated Pork Spareribs, and Crispy Pork Spareribs via UPLC-MS/MS

A method described by Lai et al. [2,9] was used to separate, identify, and quantify HAs in raw, marinated, and crispy pork spareribs via UPLC-MS/MS following extraction using QuEChERS. An ACQUITY BEH C18 column from Water Co. was used to separate 21 HA standards within 4 min by using the same gradient mobile phase of 20 mM of ammonium acetate (pH 4.5) (A) and acetonitrile (B) for the subsequent identification and quantitation of HAs in pork samples with the SRM mode and the same UPLC-MS/MS parameter including precursor ions and product ions [2,9]. The retention time and SRM detection parameters of 20 HAs and the internal standard (4,7,8-TriMeIQx) for UPLC-MS/MS are shown in Table S1.

### 2.5. Analysis of PAHs in Raw Pork Spareribs, Marinated Pork Spareribs, and Crispy Pork Spareribs via GC-MS/MS

A method described by Lai et al. [2,9] was used to separate, identify, and quantify PAHs in raw, marinated, and crispy pork spareribs by GC-MS/MS following extraction via QuEChERS. A DB-5MS capillary column from Agilent Co was employed to separate 24 PAH standards within 77 min using the same temperature programming condition for the subsequent identification and quantitation of PAHs in pork samples via GC-MS/MS with the SRM mode and the same MS parameters including precursor ions and product ions [2,9]. The retention time and SRM detection parameters of 23 PAHs and the internal standard (Triphenylene) for GC-MS/MS are shown in Table S2.

### 2.6. Method Validation of PAHs and HAs in Pork Samples

The method validation experiment of PAHs and HAs in raw pork was reported in a previous study by Lai et al. [2], and, thus, it was not performed in this study. The validation parameters such as limit of detection (LOD), limit of quantitation (LOQ), recovery, intra-day variability, and inter-day variability were determined.

### 2.7. Determination of PAH Precursors in Pork Samples via GC-MS

A method described by Lai et al. [10] was used to analyze PAH precursors including benzaldehyde, cyclohexene, trans,trans 2,4-decadienal, 2-cyclohexene-1-one, and 4,4-dimethyl-2-cyclohexene-1-one in raw, marinated and crispy pork spareribs via GC-MS/MS. In brief, a 0.5 g pork sample was poured into a 20 mL headspace vial containing 2.5 mL of water, followed by heating this mixture for 10 min at 65 °C, inserting the fiber head into the sample vial, heating for 20 min at 65 °C, and inserting into the Agilent GC-MS inlet for desorption in the injection port (260 °C) for 1 min. Then, the PAH precursors were separated using an Agilent HP-5MS column (30 m × 250 μm ID, 0.25 μm film thickness) with a temperature programming condition: the initial temperature of 40 °C was held for 4 min, raised to 50 °C at 5 °C/min, held for 2 min, increased to 120 °C at 5 °C/min, held for 3 min, raised to 260 °C at 30 °C/min, and held for 5 min. The flow rate of the carrier gas helium was 1 mL/min. The selective ion monitoring (SIM) mode with *m*/*z* at 68, 81, and 105 was used for the identification of 2-cyclohexene-1-one, trans,trans-2,4-decadienal, and benzaldehyde, respectively. Next, their contents were calculated from their respective standard curves [10]. Both cyclohexene and 4,4-dimethyl-2-cyclohexene-1-one were not quantified as they remained undetected in pork samples in GC-MS.

### 2.8. Determination of HA Precursors

#### 2.8.1. Creatine and Creatinine

A method described by Gibis & Loeffler [11] was used to determine creatine and creatinine contents in raw, marinated and crispy pork spareribs. Briefly, a 20 g pork sample was mixed with 100 mL of distilled water for subsequent homogenization for 2 min at 24,000 rpm and then stood at 18 °C for 20 min. Following filtration through filter paper, perchloric acid was added and neutralized to pH 6.5 with potassium hydroxide for the quantitation of creatine and creatinine in pork samples using the assay kits.

#### 2.8.2. Amino Acid

The individual amino acid contents in raw, marinated and crispy pork spareribs were determined using a method described by TFDA [12]. For the preparation of the derivatized amino acid standard solutions, each amino acid (37.5 mg) was dissolved in 10 mL of hydrochloric acid (1 N), followed by collecting a portion (20 μL), mixing with 100 μL of boric acid buffer solution (0.4 M) thoroughly, adding 20 μL of phthaldehyde, vortexing for 60 s, adding 9-fluorenylmethyl chloroformate (20 μL), vortexing for 30 s, adding 1280 μL of deionized water, and then mixing thoroughly. Then, a 0.5 g pork sample was mixed with 10 mL of hydrochloric acid (0.1 N) and filtered through a 0.22 μm membrane filter, followed by collecting a portion (20 μL), mixing with 100 μL of boric acid buffer solution (0.4 M), adding 20 μL of phthaldehyde, vortexing for 60 s, adding 9-fluorenylmethyl chloroformate (20 μL), vortexing for 30 s, and adding 1280 μL of deionized water for the HPLC separation of various amino acids using a Poroshell HPH-C18 column (10 cm × 3.0 mm I.D., particle size 2.7 μm) with a gradient mobile phase of 40 mM of sodium dihydrogen phosphate (pH 7.8) (A) and acetonitrile/methanol/water (45:45:10, *v*/*v*/*v*) (B) for subsequent identification and quantitation, with the former being carried out by comparing the absorption spectra and retention time of amino acid standards with peaks on the HPLC chromatogram and the latter conducted using a formula shown below [12]:

$$\mathrm{Content}\;\mathrm{of}\;\mathrm{amino}\;\mathrm{acid}\;(\text{mg/}100\;g)\;\mathrm{in}\;\mathrm{sample}=C\times \frac{V}{M}\times 10$$

where *C*: concentration of amino acid (μg/mL) obtained based on standard curves; *V*: final volume of sample extract (25 mL); and *M*: sample weight (g).

#### 2.8.3. Reducing Sugar

A method described by Chen et al. [13] was used to determine reducing sugar content in raw, marinated and crispy pork spareribs. Briefly, 0.315 g of dinitrosalicylic acid was dissolved in 50 mL of distilled water, followed by adding 10 mL of sodium hydroxide solution (0.2 g/mL) and 9.1 g of sodium potassium tartrate, diluting to 100 mL with distilled water, mixing with 0.1 mL of five glucose standard concentrations (1.25, 2.5, 5, 8, and 10 mg/mL) separately, heating at 100 °C for 10 min, cooling to room temperature, and measuring absorbance at 570 nm for the preparation of the glucose standard curve. Next, a 2 g pork sample was mixed with distilled water (10 mL), after which this mixture was shaken for 60 min and then centrifuged at 4000× *g* for 20 min. Following dilution with distilled water (50 mL), a portion (0.1 mL) was collected, mixed with dinitrosalicylic acid (1 mL), heated at 100 °C for 10 min, and cooled to room temperature, and the absorbance was measured at 570 nm for calculation of the glucose content using the linear regression equation of the glucose standard curve.

### 2.9. Principal Component Analysis (PCA)

The mean values of triplicate analyses were used to perform PCA by grouping the contents of HAs and PAHs formed under various treatment conditions. By using a Kaiser–Meyer–Olkin value of 0.80 and *p* < 0.05, the PCA was run on an Origin^®^ 2019b version 9.65 software (Northampton, MA, USA) to elucidate the relationship between the formation of HAs and PAHs in unprocessed and marinated pork spareribs as well as in soybean oil- and palm oil-fried pork spareribs at 150 °C/12 min and 190 °C/6 min.

### 2.10. Statistical Analysis

A total of 6 experimental groups, including raw pork spareribs, marinated pork spareribs, soybean oil-fried pork spareribs at 150 °C for 12 min, soybean oil-fried pork spareribs at 190 °C for 6 min, palm-oil fried pork spareribs at 150 °C for 12 min, and palm oil-fried pork spareribs at 190 °C for 6 min, were prepared and analyzed for the formation of HAs and PAHs via UPLC-MS/MS and GC-MS/MS, respectively. Each experiment was conducted in triplicate, and all of the mean data were subjected to statistical analysis using the statistical analysis system (SAS) 9.4 software (SAS Institute Inc., Cary, NC, USA) [14] for analysis of variance (ANOVA) and Duncan’s multiple range test for significance in mean comparison (*p* < 0.05). Additionally, a two-factorial design analysis was carried out using the two-way ANOVA method to elucidate the independent contributions of oil type (soybean oil and palm oil), frying conditions (150 °C/12 min and 190 °C/6 min), and their interactions (oil type × frying condition) on the formation of HAs and PAHs.

## 3. Results and Discussion

### 3.1. Separation of HA Standards by UPLC-MS/MS

**Figure 2:**
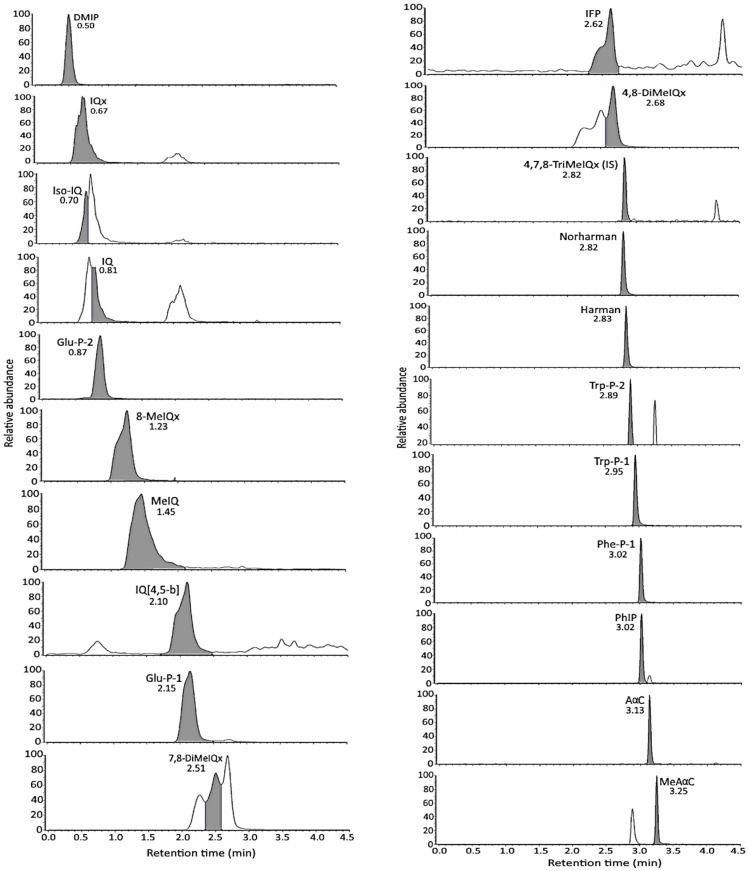
UPLC-MS/MS chromatograms of 21 HA standards as detected in selected reaction monitoring (SRM) mode. The gray portion represents peak area used for quantitation.

Following the separation condition described in the method section, a total of 21 HA standards, including internal standard 4,7,8-TriMeIQx, were separated within 4 min, with the retention times of DMIP, Glu-P-2, iso-IQ, IQ, IQx, MeIQ, Glu-P-1, 8-MeIQx, IQ[4,5-b], IFP, 7,8-DiMeIQx, 4,8-DiMeIQx, Norharman, 4,7,8-TriMeIQx, Harman, Phe-P-1, Trp-P-2, PhIP, Trp-P-1, AαC, and MeAαC being 0.50, 0.87, 0.70, 0.81, 0.67, 1.45, 2.15, 1.23, 2.10, 2.62, 2.51, 2.68, 2.82, 2.82, 2.83, 3.02, 2.89, 3.02, 2.95, 3.13, and 3.25 min, respectively (Table S1 and Figure 2). The LOD and LOQ for 20 HAs as detected by UPLC-MS/MS ranged, respectively, from 0.005 to 0.05 μg/kg and 0.01 to 0.1 μg/kg, and the recovery of 20 HAs ranged from 74.3 to 91.2% in standards and 59.4 to 104% in freeze-dried pork. Also, the coefficient of variation for intra-day variability for 20 HA standards and 20 HAs in freeze-dried pork ranged from 5.18 to 10.48% and 5.88 to 28.44%, respectively, while that for inter-day variability ranged from 6.94 to 14.72% and from 9.17 to 33.70%, respectively [2].

### 3.2. Effects of Frying Condition and Oil type on HA Formation in Crispy Pork Spareribs

Table 1 shows the HA contents in raw, marinated and pork spareribs fried in soybean oil or palm oil at 150 °C/12 min and 190 °C/6 min before and after frying. Two HAs were detected in raw pork spareribs, with Harman present at 0.16 μg/kg and Norharman at trace amounts. In the literature reports, a trace amount of Harman was also detected in raw pork [2,9], which may be due to the reaction of tryptophan with pyruvate or acetate in organisms [15]. In addition, it is also possible that the presence of Norharman and Harman in raw meat may arise from the polluted environment. Both Harman (6.68 μg/kg) and Norharman (0.62 μg/kg) remained detected in marinated pork spareribs before frying, with the contents being higher than in raw pork spareribs. As reported in a previous study [9], the addition of soy sauce to marinade for pork marinating may also contribute to the formation of Harman and Norharman. Specifically, the contents of Norharman and Harman in soy sauce were reported to be 15–71 ng/mL and 130–250 ng/mL, respectively [15]. In another study, Pan et al. [16] determined Norharman and Harman contents in 5 soy sauce products, with a range of 80.76–199.27 μg/kg for the former and 111.47–301.30 μg/kg for the latter being shown. Furthermore, the HA contents in marinated juice were shown to be higher than in marinated ground pork before frying [9], implying that soy sauce played a vital role in affecting the formation of Harman and Norharman during pork marinating.

**Figure 3:**
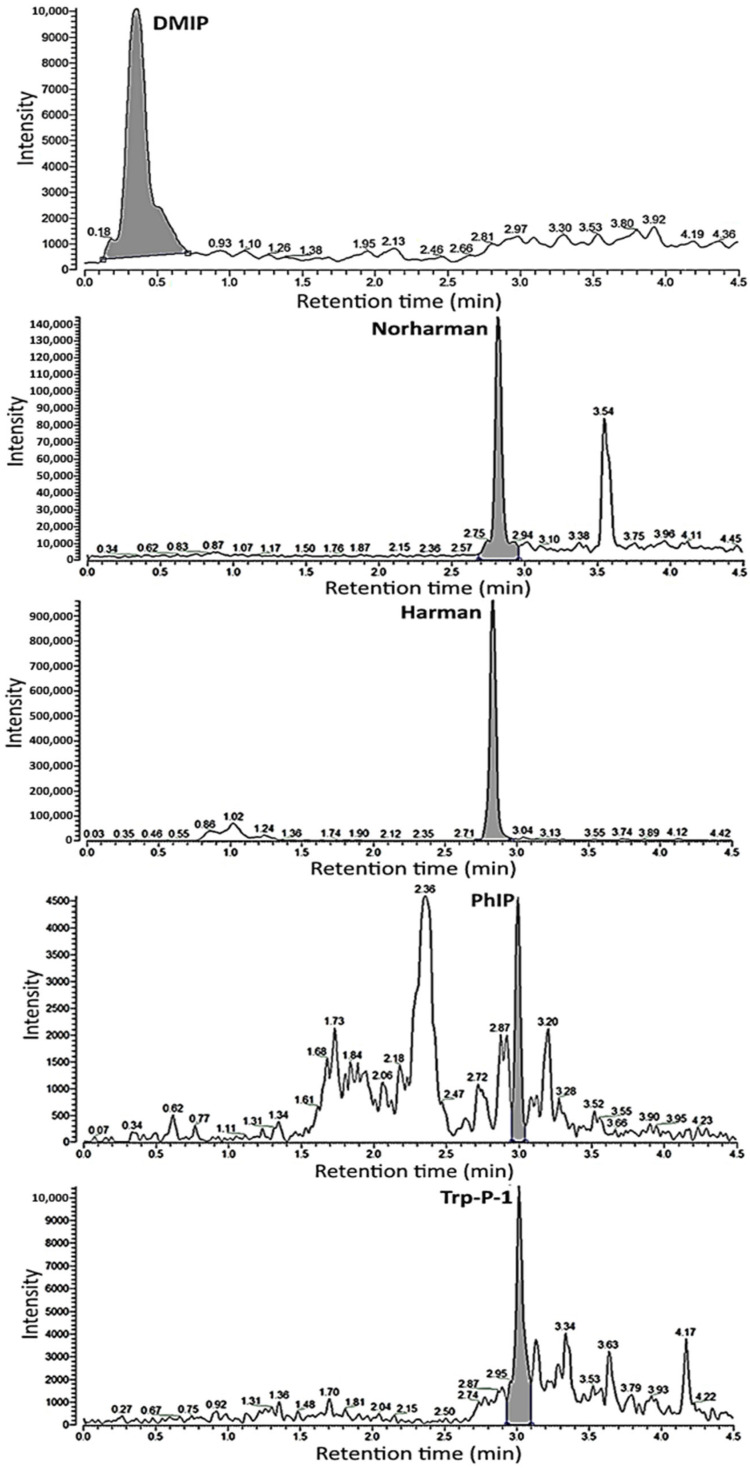
UPLC-MS/MS chromatograms of 5 HAs in crispy pork spareribs after frying in palm oil for 190 °C/6 min as detected in selected reaction monitoring (SRM) mode. The gray portion represents peak area used for quantitation.

A total of 7 HAs were detected in crispy pork spareribs fried in soybean oil at 150 °C/12 min, with Harman (14.46 μg/kg) present at the highest level, followed by Norharman (3.41 μg/kg), Trp-P-1 (1.55 μg/kg), DMIP (1.44 μg/kg), PhIP (0.91 μg/kg), and Trp-P-2 and MeAαC (both in trace amounts). Similarly, a total of 7 HAs were detected in crispy pork spareribs fried in soybean oil at 190 °C/6 min, with Harman showing the highest level (17.64 μg/kg), followed by DMIP (5.26 μg/kg), Norharman (4.54 μg/kg), Trp-P-1 (1.89 μg/kg), PhIP (1.74 μg/kg), and trace amounts of Trp-P-2 and MeAαC. Likewise, with palm oil as the frying medium at 150 °C/12 min, a total of 7 HAs were detected in the crispy pork spareribs, with Harman showing the highest level (16.87 μg/kg), followed by Norharman (4.42 μg/kg), DMIP (2.67 μg/kg), Trp-P-1 (1.72 μg/kg), PhIP (1.51 μg/kg), and trace amounts of Trp-P-2 and MeAαC. We detected 16.87, 4.42, 2.67, 1.72, and 1.51 μg/kg of Harman, Norharman, DMIP, Trp-P-1, and PhIP, respectively, and trace amounts of Trp-P-2 and MeAαC, in crispy pork spareribs fried in palm oil at 190 °C/6 min (Figure 3). Comparatively, under the same frying condition (150 °C/12 min), palm oil produced a higher level of total HAs than soybean oil. However, at 190 °C/6 min, no significant difference (*p* > 0.05) in total HAs in crispy pork spareribs was shown between palm oil and soybean oil. Moreover, a higher level of total HAs in crispy pork spareribs was shown at 190 °C/6 min than at 150 °C/12 min with soybean oil as the frying medium, revealing that frying temperature may play a more important role than frying time in affecting HA formation. Nevertheless, with palm oil as the frying medium, the total HA contents in crispy pork spareribs between 150 °C/12 min and 190 °C/6 min showed no significant difference (*p* > 0.05). In a previous study, Fan et al. [17] reported that the HA contents followed a temperature-dependent rise during the frying of beef patties, as evidenced by the total levels being 2.2, 31.4, and 69.6 μg/kg at 175 °C, 200 °C, and 225 °C for 10 min, respectively. In addition, the frying time length is also an imperative factor in affecting HA formation in beef patties [17]. By comparison, the total HAs in crispy pork spareribs were lower in our study, which can be attributed to the difference in processing condition, method, and meat commodity.

Specifically, when fried in soybean oil, the DMIP content in crispy pork spareribs rose from 1.44 μg/kg (150 °C/12 min) to 5.26 μg/kg (190 °C/6 min), while when fried in palm oil, the DMIP level increased from 2.67 μg/kg (150 °C/12 min) to 6.63 μg/kg (190 °C/6 min), which is similar to the result reported by Fan et al. [17], showing that the DMIP level in beef patties fried at 200 °C rose 11.2-fold compared to frying at 175 °C. In another report, Wang et al. [18] studied the effects of frying temperature (150, 175, 200, 225, and 250 °C) and time length (0.5, 1.0, 1.5, 2.0, and 2.5 min) on HA formation in fried pork, and the PCA showed that with high frying temperature (225–250 °C) and long frying time length (2–2.5 min), a higher HA content was generated in fried pork, with the temperature effect being more pronounced.

By comparison, under the same frying condition, especially in the low-temperature-long-time treatment (150 °C/12 min), a lower amount of total HAs in crispy pork spareribs was generated in soybean oil than in palm oil. This outcome is similar to a report by Pan et al. [19], who showed that the total HAs including 4,8-DiMeIQx, PhIP, Harman, Norharman, AαC, and MeAαC in pork floss fried in soybean oil, lard, and palm oil at 180 °C were 11.48, 14.33, and 16.83 μg/kg, respectively. In several previous studies, it was also shown that oils with higher saturation produced more free fatty acids during heating, resulting in a higher degree of oil cracking and leading to the production of more HAs [20]. As mentioned above, due to the high content of saturated fatty acids in lard and palm oil, the HA levels in pork floss fried with lard or palm oil were higher than in that fried with soybean oil [19]. In another study, Tai et al. [21] pointed out that coconut oil, containing a high amount of saturated fatty acids (lauric acid), can induce more HA formation in fish fiber when fried than in lard or soybean oil, with the contents being 92.42, 61.75, and 16.83 μg/kg, respectively. The authors postulated that the hydrolysis rate of coconut oil was faster during frying, forming a large amount of free fatty acids to accelerate the degradation rate of lipids, resulting in HA formation. In a study dealing with meatball frying in sunflower oil, corn oil, hazelnut oil, rapeseed oil, olive oil, and commercial oil blends, the lowest MeIQx content (30.43 μg/kg) was shown in hazelnut oil, while the highest MeIQx level was shown in commercial oil blends (43.71 μg/kg), with the saturated fatty acid levels before and after frying being 30.32% and 31.05%, respectively [22]. In contrast, many studies have shown that highly unsaturated oils, such as soybean oil or sesame oil, should be more susceptible to HA formation than saturated oil such as lard, as the former is rich in polyunsaturated fatty acids, which can produce reactive oxygen species for the subsequent degradation of Amadori compounds and formation of Maillard reaction product intermediates 1-deoxysone and 3-deoxysone, leading to HA production [10,22,23]. Currently, there are two possible reasons to explain why fat can promote HA formation. (1) Physical reaction: fat, as a heat transfer medium, can affect HA generation by accelerating the heat transfer efficiency. (2) Chemical reaction: fat oxidation can generate more free radicals participating in the Maillard reaction, leading to the formation of certain pyrazines and pyridines for the subsequent generation of polar HAs. Conversely, nonpolar HAs are generally produced by amino acid cleavage and are less directly related to fat oxidation [24]. With the exception of DMIP and PhIP, the HAs detected in our study are mainly nonpolar HAs (Norharman, Harman, Trp-P-2, Trp-P-1, and MeAαC), and, thus, it can be inferred that the degree of fat unsaturation may play a less significant role in HA formation in crispy pork spareribs during frying.

### 3.3. Content Changes of HA Precursors in Crispy Pork Spareribs during Processing

Table 2 shows the effect of oil type and frying condition on the contents of individual amino acids (mg/g) in crispy pork spareribs. A total of 6.61 mg/g was shown in raw pork spareribs, while a total of 9.87 mg/g was found in the marinated pork spareribs. As mentioned above, soy sauce may contribute to HA formation such as Harman and Norharman in marinated juice. After frying at 150 °C/12 min and 190 °C/6 min, the total amino acid content of crispy pork spareribs fried in soybean oil was 9.26 and 8.78 mg/g, respectively, as well as 9.07 and 8.59 mg/g, with palm oil as the frying medium. Also, the highest amount of total amino acids was shown in pork spareribs fried in soybean oil at 150 °C/12 min. It may be inferred that the lower the amino acid content, the more the amino acids participate in the Maillard reaction, and the greater the generation of HAs. Moreover, with soybean oil or palm oil as the frying medium, the total amino acid content in crispy pork spareribs fried at 190 °C/6 min was lower than that at 150 °C/12 min, revealing that the former frying condition is more likely to promote HA formation, as shown in Table 1.

**Figure 4:**
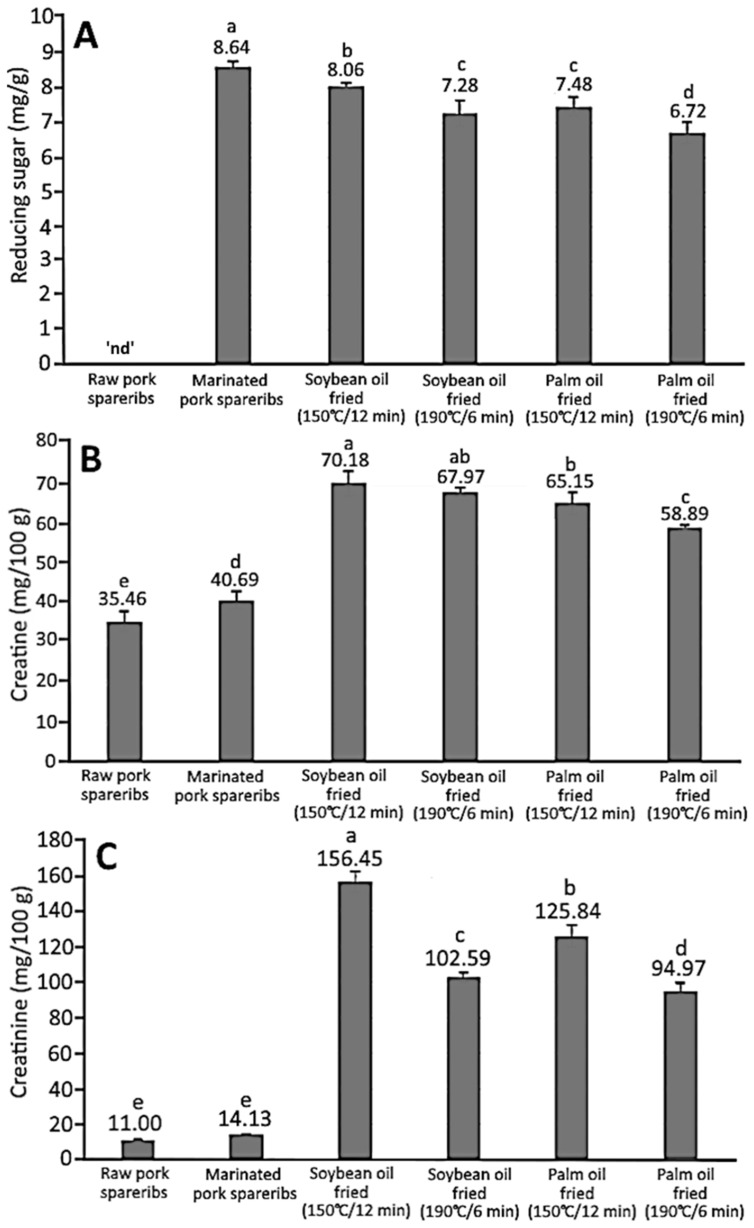
Contents of HA precursors including reducing sugar (**A**), creatine (**B**), and creatinine (**C**) in crispy pork spareribs as affected by oil type and processing condition. ‘nd’ denotes that the reducing sugar value is not detected. The different small letters (a–e) on each bar represent significantly different values (*p* < 0.05) within each bar graph.

Figure 4 shows the effects of oil type and frying condition on the contents (mg/g) of reducing sugar, creatine, and creatinine in crispy pork spareribs. It was shown that the reducing sugar content of pork spareribs rose to 8.64 mg/g after marinating and was reduced to 8.06 and 7.28 mg/g, respectively, following frying in soybean oil at 150 °C/12 min and 190 °C/6 min, as well as to 7.48 and 6.72 mg/g in palm oil. However, the reducing sugar content in pork spareribs fried in soybean oil at 150 °C/12 min did not show a declining trend, which may be due to the rate of reducing sugar formation being greater than the rate of participation in the Maillard reaction. Also, the content of reducing sugars was lower than that in marinated pork spareribs, indicating that more reducing sugars were consumed during the frying process by participating in the Maillard reaction and formation of HAs.

When muscle tissue is heated, creatine can be converted to creatinine, a precursor of the formation of the imidazole ring. Therefore, the concentration of creatine or creatinine is positively correlated with the content of mutagenic compounds, such as HAs produced during heat treatment [25]. According to the results in Figure 4, the content of creatine in crispy pork spareribs after frying was higher than that in raw pork spareribs (35.46 mg/100 g) and marinated pork spareribs (40.69 mg/100 g), as evidenced by a higher content level of 70.18 and 67.97 mg/100 g following frying at 150 °C/12 min and 190 °C/6 min in soybean oil, respectively, as well as 65.15 and 58.89 mg/100 g in palm oil. Compared to creatine, the creatinine content was much lower in raw pork spareribs (11.00 mg/100 g) and marinated pork spareribs (14.13 mg/100 g), which should be caused by the partial conversion of creatine. However, the creatinine contents of pork spareribs fried in soybean oil at 150 °C/12 min and 190 °C/6 min were raised to 156.45 and 102.59 mg/100 g, respectively, while those fried in palm oil at 150 °C/12 min and 190 °C/6 min were 125.84 and 94.97 mg/100 g, respectively. Apparently, more creatine was converted to creatinine in crispy pork spareribs for subsequent reaction with amino acid/reducing sugar for HA formation during frying. Moreover, the creatinine content in crispy pork spareribs fried at a high temperature within a short time (190 °C/6 min) was lower than that of spareribs fried at a low temperature over a long time (150 °C/12 min), implying that the former condition could facilitate the creatinine reaction. Additionally, under the same frying condition, it was found that frying with palm oil resulted in a lower creatinine content than frying with soybean oil, indicating that more creatinine participated in the HA production, leading to a higher level of HAs in crispy pork spareribs with palm oil as the frying medium. As a high amount of creatinine was shown in fried pork spareribs, it may be postulated that the conversion rate of creatine to creatinine was higher than the reaction rate of creatinine, a precursor of imidazole formation.

It is worth pointing out that IQ, a probable human carcinogen, remained undetected in fried crispy pork spareribs, while some other HAs, including PhIP, Trp-P-1, Trp-P-2, and MeAαC, all of which are possible human carcinogens, were present in small amounts. Nevertheless, crispy pork spareribs should be consumed with caution, as excessive intake may cause adverse effects on human health.

### 3.4. Separation of PAH Standards via GC-MS/MS

**Figure 5:**
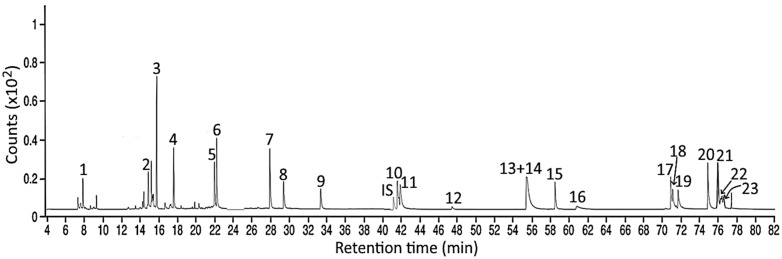
GC-MS/MS chromatograms of 24 PAH standards including internal standard (IS) as detected in selected reaction monitoring (SRM) mode. Peaks: 1, NaP; 2, AcPy; 3, AcP; 4, Flu; 5, Phe; 6, Ant; 7, FL; 8, Pyr; 9, BcF; IS, 4,7,8-TriMeIQx; 10, BaA; 11, CHR; 12, MCH; 13, BbF; 14, BjF; 15, CcdP; 16, BaP; 17, IP; 18, DBahA; 19, BghiP; 20, DBalP; 21, DBaeP; 22, DBaiP; and 23, DBahP.

Following the separation condition described in the method section, a total of 24 PAH standards, including internal standard Triphenylene, were separated within 77 min, with the retention times of NaP, AcPy, AcP, Flu, Phe, Ant, FL, Pyr, BcF, Triphenylene, BaA, CHR, MCH, BbF, BjF, CcdP, BaP, IP, DBahA, BghiP, DBalP, DBaeP, DBaiP, and DBahP being 7.90, 14.5, 15.6, 17.6, 21.8, 22.1, 27.9, 29.5, 33.6, 41.1, 42.0, 41.6, 47.5, 55.8, 55.8, 58.3, 61.2, 70.8, 71.0, 71.6, 74.9, 75.9, 76.5, and 76.8 min, respectively. However, both BbF and BjF were overlapped (Table S2 and Figure 5).

The LOD and LOQ for 22 PAHs as detected using GC-MS/MS ranged, respectively, from 0.03 to 0.5 μg/kg and 0.1 to 1.5 μg/kg, and the recovery of 22 PAHs ranged from 84.6 to 107.6% in standards and from 80.1 to 101.1% in freeze-dried pork. Also, the coefficient of variation for intra-day variability for 22 PAH standards and 22 PAHs in freeze-dried pork ranged from 5.03 to 10.57% and from 6.74 to 15.60%, respectively, while that for inter-day variability ranged from 9.12 to 17.25% and from 11.91 to 20.61% [2].

### 3.5. Effects of Frying Condition and Oil Type on PAH Contents in Crispy Pork Spareribs

Table 3 shows the PAH contents in raw and marinated pork spareribs, with 3 PAHs including Pyr (3.52 μg/kg), DBaeP (trace), and DBaiP (trace) detected in the former and 7 PAHs including Pyr (10.66 μg/kg), BaA (0.87 μg/kg), BbF (1.70 μg/kg), CcdP (1.37 μg/kg), BjF (trace), DBaeP (trace), and DBaiP (trace) detected in the latter. The presence of PAHs in raw pork spareribs may be associated with environmental factors including polluted air, water, and soil [26]. Interestingly, a substantial rise of 11.09 μg/kg was shown in raw pork spareribs following marinating, which may be due to the presence of PAHs in marinades such as soy sauce and spice powder. This phenomenon was also observed in a previous study dealing with PAH formation in ground pork before and after marinating [9]. As reported by Lai et al. [9], the addition of a variety of marinades in various amounts can affect the type and content of PAHs formed in marinated pork during processing.

**Figure 6:**
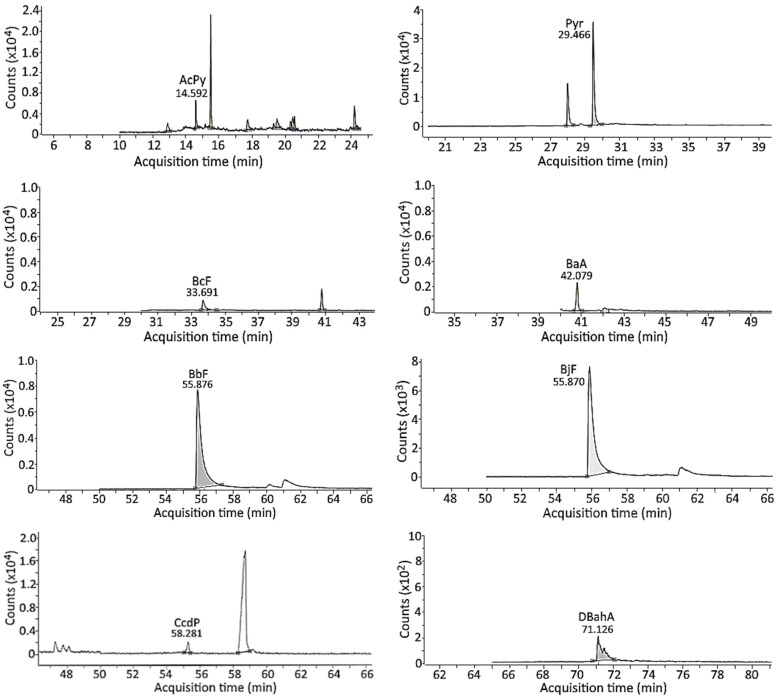

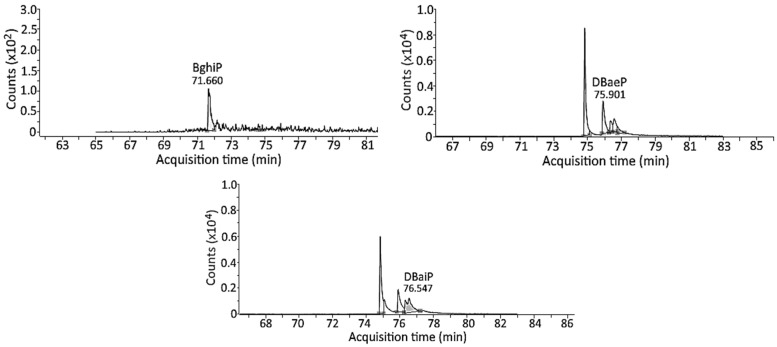
GC-MS/MS chromatograms of 11 PAHs in crispy pork spareribs after frying in soybean oil for 150 °C/12 min as detected via selected reaction monitoring (SRM). The gray portion represents peak area used for quantitation.

A total of 12 PAHs were detected in crispy pork spareribs fried in soybean oil at 150 °C/12 min (Figure 6), with the highest content being CcdP (32.78 μg/kg), followed by Pyr (22.36 μg/kg), BjF (10.15 μg/kg), DBaiP (5.32 μg/kg), BbF (5.27 μg/kg), BaA (3.85 μg/kg), DBahA (1.86 μg/kg), BcF (1.56 μg/kg), BghiP (1.27 μg/kg), DBaeP (0.59 μg/kg), AcPy (0.08 μg/kg), and AcP (trace). Similarly, a total of 12 PAHs were detected in crispy pork spareribs fried in soybean oil at 190 °C/6 min, with the highest level being CcdP (27.92 μg/kg), followed by Pyr (20.87 μg/kg), BjF (10.15 μg/kg), DBaiP (5.37 μg/kg), BbF (4.78 μg/kg), BaA (3.87 μg/kg), DBahA (1.85 μg/kg), BcF (1.59 μg/kg), BghiP (1.27 μg/kg), DBaeP (0.55 μg/kg), AcPy (0.07 μg/kg), and AcP (trace). But for the PAH contents in crispy pork spareribs fried in palm oil at 150 °C/12 min, a total of 11 PAHs were detected, with the highest content being CcdP (24.33 μg/kg), followed by Pyr (17.89 μg/kg), BjF (9.65 μg/kg), DBaiP (5.28 μg/kg), BbF (4.77 μg/kg), BaA (3.84 μg/kg), DBahA (1.86 μg/kg), BcF (1.56 μg/kg), BghiP (1.27 μg/kg), and trace amounts of AcPy and DBaeP. A similar outcome was shown for the PAH contents in crispy pork spareribs fried in palm oil at 190 °C/6 min (Table 3). By comparison, under the same frying condition, a higher level of total PAHs in pork spareribs fried in soybean oil was shown than in palm oil, indicating that the highly unsaturated soybean oil may be more liable to PAH formation during frying [9,10]. Furthermore, with soybean oil or palm oil as the frying medium, the frying condition at 150 °C/12 min produced a higher level of total PAHs than at 190 °C/6 min, implying that a longer frying time should be more important than frying temperature in affecting PAH formation in pork spareribs. A higher level of CcdP generated compared to Pyr in pork spareribs during frying in soybean oil or palm oil may be caused by the conversion of four-ringed Pyr upon a cycloaddition reaction into five-ringed CcdP [27].

In several previous studies, Hu et al. [28] explored the effect of frying time on PAH formation in sunflower oil during frying, and the total contents of BaA, CHR, BkFL, BbF, BaP, IP, DBahA, and BghiP followed a time-dependent rise and reached 27.82 ppb following 72 h of frying. It is worth pointing out that the highly toxic BaP was detected at 4.00 μg/kg. In another study dealing with the analysis of PAH levels in edible oil during frying, high-molecular-weight PAHs (>5 rings) of samples fried for 45 min were produced in amounts 1.9-fold higher than those of samples fried for 15 min [29]. This result is in accordance with our study, showing that a total of seven high-molecular-weight PAHs, including BbF, BjF, CcdP, BghiP, DBahA, DBaeP, and DBaiP, were detected in fried pork spareribs. Similarly, An et al. [30] fried bread sticks for 32 h and reported that the total contents of 16 PAHs, including Nap, Acy, AcPy, Flu, Phe, Ant, FL, Pyr, BaA, CHR, BbF, BkFL, BaP, BghiP, IP, and DBahA, rose from 18 to 57 ppb. In a recent study, Xu et al. [31] illustrated that under different frying conditions, the interaction between food and oil in the frying system can affect PAH formation, with frying method, condition, food composition, and degree of oil unsaturation playing a crucial role. In addition, the presence of antioxidants in the oil system may reduce PAH formation. For instance, Siddique et al. [32] compared the effects of frying methods on PAH formation in various parts of rabbit meat. Only Fluorene was detected in all samples, with the highest level of 90–170 ppb being observed for the sample with 160 mL of oil fried at 90 °C for 4 min, followed by 40–100 ppb (sample stir-fried in 5 mL of oil at 120 °C for 8 min) and 10–50 ppb (sample pan-fried at 90 °C for 8 min without oil). Ge et al. [33] studied the effect of frying (200 mL of rapeseed oil, 226–228 °C, 5 min) and pan-frying (50 mL of rapeseed oil, 226–228 °C, 3 min) on PAH formation in fried beef, pork, chicken, and duck meat, and the results showed that with the exception of beef, the PAH contents in fried samples were higher than those in pan-fried samples, indicating that frying may speed up PAH formation. In another study, Lee et al. [34] studied the effect of air frying (180 °C, 35 min, no oil) and conventional frying (226–228 °C, 3 min, 200 mL oil) on PAH4 (BaA, CHR, BbF, and BaP) formation in chicken meat, and the PAH4 contents in air-fried chicken (2.71 ppb) were significantly lower than those in conventional fried chicken (13.7 ppb). Siddique et al. [35] also compared the effects of microwave frying and traditional pan-frying, stir-frying, and deep-frying on PAH formation in rabbit meat and reported that the PAH contents in rabbit meat samples following microwave frying were significantly lower than those following pan-frying, stir-frying, and deep-frying, with the levels being 0.67–1.27 ppb, 10–50 ppb, 40–100 ppb, and 90–170 ppb, respectively. Thus, it is possible to reduce PAH formation by using some other types of frying methods such as microwave frying or air frying.

In addition to the impact of the frying method and condition on PAH formation in fried meat products, the formation of PAH in fumes generated during frying cannot be ignored. For example, Chiang et al. [36] studied the fume produced by cooking lard, soybean oil, and peanut oil and found that all samples contained DBahA and BaA, with a level of 1.9 and 2.2 μg/m^3^ in lard fumes, respectively, as well as 2.1 and 2.3 μg/m^3^ in soybean oil fume and 1.8 and 1.3 μg/m^3^ in peanut oil fume, while the highly toxic BaP levels in soybean oil and peanut oil fumes were 19.6 and 18.3 μg/m^3^, respectively, revealing an increased risk of lung cancer in women exposed to these cooking oil fumes. Chen & Chen [37] also reported that PAHs such as naphthalene can be produced in the smoke from model lipid during heating through a reaction between 1,3-butadiene (from linoleic acid oxidation) and benzaldehyde (from cyclohexene oxidation) for a subsequent Diels–Alder cycloaddition reaction.

### 3.6. Content Changes of PAH Precursors in Crispy Pork Spareribs during frying

Table 4 shows the effect of oil type and frying condition on the contents of PAH precursors in fried pork spareribs. A total of 4 fatty acid cracking products were determined, including 4,4-dimethyl-2-cyclohexene-1-one, 2-cyclohexene-1-one, benzaldehyde, and trans,trans-2,4-decadienal. As mentioned above, benzaldehyde may be generated through the oxidation of cyclohexene for subsequent reaction with linoleic acid degradation products such as 1,3-butadiene from soybean oil through a Diels–Alder cycloaddition for PAH formation [37]. For 2-cyclohexene-1-one, it may be produced from the oxidation and degradation of linolenic acid in soybean oil during heating for subsequent reaction with C4 structure substances for naphthalene formation through a Diels–Alder reaction. Likewise, trans,trans-2,4-decadienal formed from linoleic acid may react with 2-butene (dienophile compounds) to generate 4-penty-2,3-dimethyl-benzoic acid for subsequent reaction with C4-containing compounds to produce naphthalene. Additionally, 4,4-dimethyl-2-cyclohexene-1-one may produce derivatives of naphthalene such as 1,2-dimethyl-naphthalene, 1,3-dimethyl-naphthalene, and decahydro-1,6-dimethyl-naphthalene during heating [37]. The PAH precursor contents in raw and marinated pork spareribs were 2-cyclohexene-1-one (1.80 μg/kg) and benzaldehyde (5.21 μg/kg) for the former, as well as 0.76 μg/kg and 12.95 μg/kg for the latter. Following the frying of pork spareribs in soybean oil at 150 °C/12 min, the benzaldehyde content reached a plateau (275.09 μg/kg), followed by 2-cyclohexene-1-one (10.58 μg/kg) and trans,trans-2,4-decadienal (1.74 μg/kg). However, with soybean oil as the frying medium at 190 °C/6 min, benzaldehyde was also produced at the highest level (115.53 μg/kg), followed by trans,trans-2,4-decadienal (5.76 μg/kg), 2-cyclohexene-1-one (5.14 μg/kg), and 4,4-dimethyl-2-cyclohexene-1-one (0.60 μg/kg). Comparatively, a much higher level of total PAH precursors was generated in pork spareribs fried in soybean oil at 150 °C/12 min than at 190 °C/6 min. A similar trend was shown for the total PAH precursor contents in pork spareribs fried in palm oil at 150 °C/12 min and 190 °C/6 min. However, under the same frying condition, the total PAH precursor contents were much lower by 246.0 μg/kg at 150 °C/12 min and 96.99 μg/kg at 190 °C/6 min with palm oil as the frying medium compared to soybean oil. Thus, lower production of total PAHs was shown in pork spareribs fried in palm oil (Table 3). A similar outcome was reported by Pan et al. [19], showing that the polyunsaturated fatty acid content of pork floss with soybean oil as the frying medium was significantly higher (*p* < 0.05) than that of pork floss fried in lard or palm oil. Obviously, a higher degree of oil unsaturation plays a vital role in the generation of more PAHs during frying. However, in another study, Gong et al. [38] reported that the total PAH contents in soybean oil- and palm oil-fried dough sticks were 9.67 μg/kg and 12.48 μg/kg, respectively, which was different from our experiment, probably due to the total PAH contents in fresh palm oil being higher than in fresh soybean oil, resulting in a higher level of total PAHs in palm oil-fried samples. Collectively, the total PAH contents were reduced by frying pork spareribs in palm oil at 190 °C/6 min or 150 °C/12 min in our study.

For dietary PAH safety, the EU proposes that the maximum residual level of PAH4 (BaA, CHR, BbF, and BaP) in edible oil is 10 μg/kg, while that of BaP is 2 μg/kg and that of PAH4 in barbecue meat products is 12 μg/kg [39]. In Taiwan, TFDA proposes a BaP limit of 2 μg/kg for edible oil and smoked meat products [40]. As the total PAH4 content in all treatment groups in this study was lower than 10 μg/kg, with BaP remaining undetected, the consumption of crispy pork spareribs prepared in this study should be quite safe. Nevertheless, like HAs, the excessive intake of crispy pork spareribs should be avoided to prevent adverse health effects.

In the above discussion, the effects of oil type and frying condition (temperature/time length) on the formation of HAs and PAHs in crispy pork spareribs were adequately elucidated. In addition, the high contents of individual HAs such as Harman and Norharman and PAHs such as CcdP and Pyr were explained. For individual PAHs, CcdP was generated at a higher level than Pyr in pork spareribs during frying in palm oil or soybean oil. As mentioned above, it may be postulated that Pyr, a PAH containing four rings, can undergo cycloaddition to promote the formation of CcdP, a PAH containing five rings [27]. The formation of Harman and Norharman may be due to the reaction of tryptophan with pyruvate or acetate in pork. In addition, the incorporation of soy sauce into marinade may contribute to the formation of Harman and Norharman [9]. At the same oil type, the effects of frying temperature/time length on the formation of HAs and PAHs in fried pork spareribs were discussed. Similarly, at the same frying temperature/time length, the effect of oil type on the generation of HAs and PAHs in fried pork spareribs was also discussed. Most importantly, the precursors associated with HA and PAH formation in pork spareribs were determined to assist in elucidating the formation mechanism of HAs and PAHs.

### 3.7. Factorial Design Analysis

A two-factorial analysis of HA and PAH formation as affected by frying condition and oil type was conducted using the two-way ANOVA method, and the results are shown in Table 5. A *p*-value of 0.0007 and 0.0097 was shown for frying condition and oil type, respectively, indicating that these factors individually had a significant impact on HA formation in crispy pork spareribs, with the frying condition being more impactful than oil type. In addition, the interaction between frying condition and oil type was insignificant, as a *p*-value of 0.0141 was found. This outcome is in agreement with the results shown in Table 1, revealing that HAs were more susceptible to formation in the high-temperature short-time treatment (190 °C/6 min) than in the low-temperature long-time treatment (150 °C/12 min) with the same oil type. However, under the same frying condition with palm oil as the frying medium, a higher level of total HAs was shown compared to soybean oil. Like HAs, the interaction between frying condition and oil type showed no significant impact on PAH formation in crispy pork spareribs (*p*-value, 0.0627), but the oil type (*p*-value, <0.0001) could more significantly affect PAH formation than the frying condition (*p*-value, 0.0010). This outcome is in line with the results shown in Table 3, suggesting that PAH is more liable to formation in soybean oil than in palm oil under the same frying condition. However, with the same oil type, a higher level of total PAHs was found for the low-temperature long-time (150 °C/12 min) treatment compared to the high-temperature short-time (190 °C/6 min) treatment.

### 3.8. Correlation between HA and PAH Formation in Crispy Pork Spareribs

**Figure 7:**
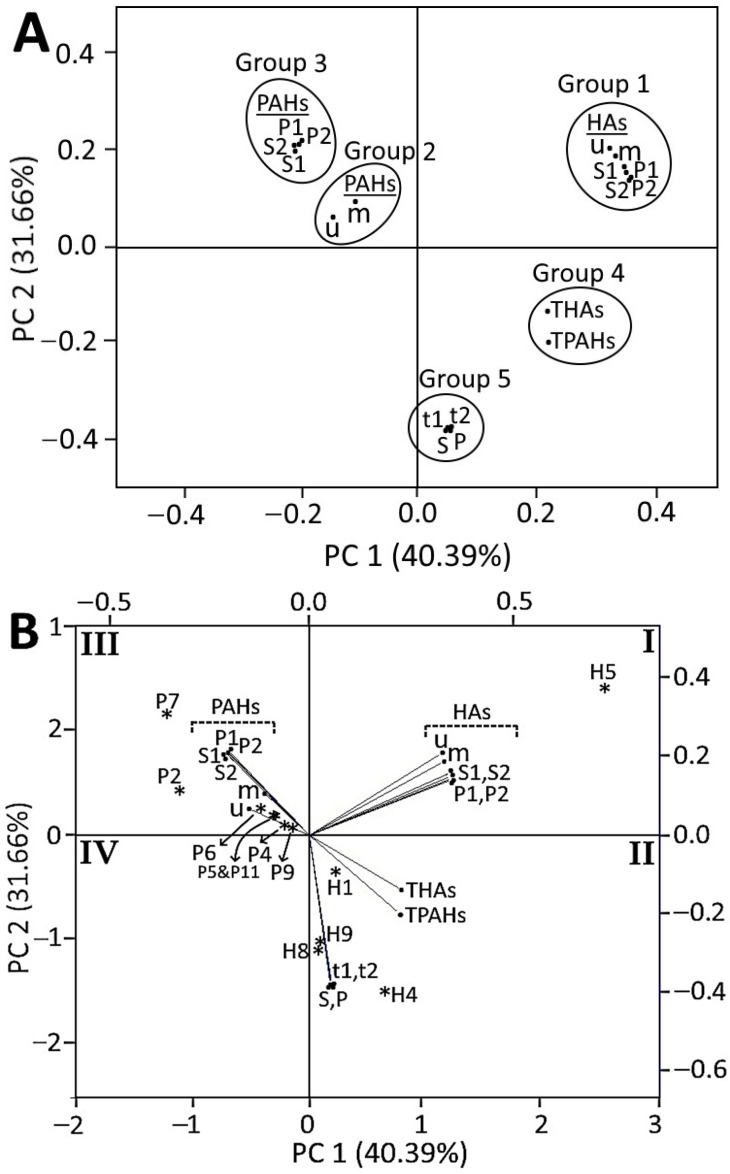
Principal component analysis illustrating score plot (**A**) and biplot containing loading plot and score plot (**B**) for HA and PAH formation in crispy pork spareribs as affected by oil type and frying condition. u and m represent the amount of HAs or PAHs formed in unprocessed raw pork spareribs and marinated raw pork spareribs before frying, respectively; S1 and S2 represent the amount of HAs or PAHs formed in soybean oil-fried pork spareribs at 150 °C/12 min and 190 °C/6 min; P1 and P2 represent the amount of HAs or PAHs formed in palm oil-fried pork spareribs at 150 °C/12 min and 190 °C/6 min; S and P represent the amount of HAs or PAHs formed regardless of temperature/time length during frying of pork spareribs in soybean oil and palm oil; and t1 and t2 represent the amount of HAs or PAHs formed regardless of oil type during frying of pork spareribs at 150 °C/12 min and 190 °C/6 min. The dot (•) symbol denotes principal component data for the formation of HAs or PAHs in crispy pork spareribs during frying. The asterisk (∗) symbol indicates the highly formed individual HA or PAH as affected by different oil types and processing conditions. H1, DMIP; H4, Norharman; H5, Harman; H8, PhIP; H9, Trp-P-1; P2, Pyr; P4, BaA; P5, BbF; P6, BjF; P7, CcdP; P9, DBahA; P11, DBaiP.

The PCA performed for the formation of HAs and PAHs in pork spareribs as affected by different oil types and processing conditions is shown in Figure 7. A total of three principal components (PC 1–3) was obtained with an eigenvalue of correlation matrix >1, revealing that PC 1–3 can mostly describe all of the data sets. Among PC 1–3, PC 1 (40.39%) and PC 2 (31.66%) contributed to the maximum total variation of 72.05% in HA and PAH formation. The score plot shown in Figure 7A shows that the experimental mean content data of HA and PAH formation can be successfully grouped into five groups depending on their extent of separation within the four quadrants. Group 1 represents various HAs formed under six different treatments, including unprocessed raw pork spareribs (u), marinated pork spareribs (m), soybean oil-fried pork spareribs at 150 °C/12 min (S1), soybean oil-fried pork spareribs at 190 °C/6 min (S2), palm oil-fried pork spareribs at 150 °C/12 min (P1), and palm oil-fried pork spareribs at 190 °C/6 min (P2). On the other hand, various PAHs formed under the same six different treatments as above were grouped into two groups, with group 2 encompassing PAH formation in “u” and “m” treatments and group 3 constituting PAH formation in the remaining 4 treatments (S1, S2, P1, and P2). A distinct separation of group 1 from groups 2 and 3 implied that HAs and PAHs are formed by different mechanisms. Obviously, HAs can be generated through the reaction of amino acids from soy sauce and pork spareribs with reducing sugar and creatine/creatinine, while benzaldehyde, 2-cyclohexene-1-one, and trans,trans-2,4-decadienal are responsible for PAH formation during frying. Also, the PAH contents in “u” and “m” treatments in group 2 were significantly separated from those in the other four frying treatments (S1, S2, P1, and P2) in group 3, indicating that PAHs were formed at relatively higher levels in soybean oil- or palm oil-fried pork spareribs compared to HAs. In other words, PAHs are more susceptible to formation than HAs during frying. The total contents of HAs (THAs) and PAHs (TPAHs) generated under different treatments regardless of oil type and processing condition were grouped into group 4, suggesting that both are formed in significant amounts (0.16–25.97 μg/kg for THAs and 3.52–85.10 μg/kg for TPAHs) in all studied conditions (Table 1 and Table 3). Likewise, the total contents of HAs and PAHs generated in soybean oil (S) and palm oil (P) regardless of temperature/time length as well as those of HAs and PAHs formed in fried pork spareribs at 150 °C/12 min (t1) and 190 °C/6 min (t2) regardless of oil type were grouped into group 5, corroborating that both HAs and PAHs are significantly formed in all treatment conditions.

Figure 7B depicts the biplot containing both loading plots and score plots with a large angle between the loading plots shown for group 1 and group 2 plus group 3 as well as a marked angle between THAs and TPAHs in group 4, further confirming that HAs and PAHs are formed by different mechanisms. It is worth mentioning that the larger the angle between the loading plots, the smaller the correlation in the formation of HAs and PAHs. However, the loading plots of total HAs or PAHs formed in soybean oil and palm oil (S and P), as well as those generated at 150 °C/12 min and 190 °C/6 min (t1 and t2) in group 5, were overlapped, suggesting that the difference in the levels of HAs or PAHs formed under different processing conditions remains low as shown by a percentage deviation from the mean value of total HA or PAH contents ranging from 1.5 to 15.8% for the former and from 3.8 to 12.9% for the latter. Moreover, the inclination of loading plots of HA formation shown in group 1 toward the PC 1 direction and that of PAH formation shown in group 2 plus group 3 toward the PC 2 direction indicated their respective influence on the two principal components of PC 1 (40.39%) and PC 2 (31.66%).

Figure 7B also shows the score plots of individual HA or PAH formations under different processing conditions marked with asterisk symbols in quadrants I-III, with five asterisks bearing the labels H1, H4, H5, H8, and H9 in quadrants I and II representing the DMIP, Norharman, Harman, PHIP, and Trp-P-1 formation and seven asterisks bearing the labels P2, P4, P5 and P11, P6, P7, and P9 in quadrant III denoting Pyr, BaA, BbF and DBaiP, BjF, CcdP, and DBahA formation. Also, the position of five asterisks in quadrants 1 and II moving toward the vertical zero line in Figure 7B shows the influence of five HAs formed on PC 1 in the following order: Harman (H5) > Norharman (H4) > DMIP (H1) > Trp-P-1 (H9) > PhIP (H8). Likewise, the position of seven asterisks in quadrant III moving toward the horizontal zero line implies the influence of seven PAHs formed on PC 2 in the following order: CcdP (P7) > Pyr (P2) > BjF (P6) > BbF and DBaiP (P5 and P11) > BaA (P4) > DBahA (P9). Furthermore, the above trend also indicated the levels of the top five HA and top seven PAH compounds formed under different treatment conditions, which conforms well with the respective row-wise total content of HAs or PAHs regardless of oil type and processing condition, as evident from Table 1 and Table 3. Thus, the biplot in Figure 7B presents a comprehensive grouping and correlation for HA and PAH formation as affected by oil type and processing condition. Overall, the PCA confirmed the conclusion drawn from the results that significant amounts of both HAs and PAHs were formed in pork spareribs during frying through different mechanisms, with the latter being more prone to formation with CcdP and Pyr dominating in PAH formation and Harman and Norharman dominating in HA formation.

## 4. Conclusions

In conclusion, the formation of HAs and PAHs in pork spareribs during frying was monitored via UPLC-MS/MS and GC-MS/MS, respectively. Both HAs and PAHs in pork spareribs during frying at 150 °C/12 min or 190 °C/6 min followed a time- and temperature-dependent rise, with PAHs being more susceptible to formation than HAs. Soybean oil produced a higher content of total PAHs and a lower amount of total HAs than palm oil, which can be attributed to the difference in the degree of oil unsaturation and the precursor content changes. A PCA confirmed the conclusion drawn from the results that HAs and PAHs in crispy pork spareribs are formed by different mechanisms during frying, while a two-factorial analysis revealed that the interaction between frying condition and oil type was insignificant for the formation of HAs and PAHs in crispy pork spareribs. Furthermore, the limitation of this study is that it mainly focuses on the formation of PAHs and HAs in pork spareribs as affected by different frying conditions. Future research is needed to reduce the formation of PAHs and HAs in pork spareribs or related fried meat products through the incorporation of additives or spices.

## Figures and Tables

**Table 1:** HA contents (μg/kg) in crispy pork spareribs as affected by oil type and processing condition ^1,2^.

HA	Raw Pork Spareribs	Marinated Pork Spareribs	Crispy Pork Spareribs			
			Soybean Oil		Palm Oil	
			150 °C/12 min	190 °C/6 min	150 °C/12 min	190 °C/6 min
DMIP	nd ^3^	nd	1.44 ± 0.06 ^d^	5.26 ± 0.46 ^b^	2.67 ± 0.42 ^a^	6.63 ± 0.45 ^c^
Norharman	trace ^4^	0.62 ± 0.32 ^c^	3.41 ± 0.37 ^b^	4.54 ± 0.31 ^a^	4.42 ± 0.34 ^a^	4.55 ± 0.16 ^a^
Harman	0.16 ± 0.02 ^d^	6.68 ± 1.92 ^c^	14.46 ± 0.74 ^b^	17.64 ± 0.62 ^a^	16.87 ± 1.04 ^a^	17.91 ± 1.45 ^a^
Trp-P-2	nd	nd	trace	trace	trace	trace
PhIP	nd	nd	0.91 ± 0.09 ^b^	1.74 ± 0.03 ^a^	1.51 ± 0.03 ^a^	1.53 ± 0.25 ^a^
Trp-P-1	nd	nd	1.55 ± 0.04 ^c^	1.89 ± 0.06 ^a^	1.72 ± 0.06 ^a^	1.98 ± 0.05 ^b^
MeAαC	nd	nd	trace	trace	trace	trace
Total	0.16 ± 0.02 ^d^	7.30 ± 1.60 ^c^	20.34 ± 0.96 ^b^	25.81 ± 0.86 ^a^	24.52 ± 0.76 ^a^	25.97 ± 1.65 ^a^

^1^ Data are presented as mean ± standard deviation of 9 determinations. ^2^ Data with different small letters (^a–d^) in the same row are significantly different (*p* < 0.05). ^3^ Not detected. ^4^ LOQ ≥ HAs levels ≥ LOD.

**Table 2:** Amino acid (AA) contents (mg/g) in crispy pork spareribs as affected by oil type and frying condition ^1,2^.

AA	Raw Pork Spareribs	Marinated Pork Spareribs	Crispy Pork Spareribs			
			Soybean Oil		Palm Oil	
			150 °C/12 min	190 °C/6 min	150 °C/12 min	190 °C/6 min
Aspartic acid	0.56 ± 0.04	0.70 ± 0.04	0.61 ± 0.05	0.65 ± 0.02	0.69 ± 0.02	0.56 ± 0.01
Glutamic acid	0.91 ± 0.05	1.06 ± 0.04	1.09 ± 0.02	1.18 ± 0.02	1.00 ± 0.02	0.87 ± 0.02
Serine	0.30 ± 0.01	0.42 ± 0.03	0.45 ± 0.04	0.32 ± 0.01	0.37 ± 0.01	0.35 ± 0.02
Histidine	0.06 ± 0.02	0.04 ± 0.01	0.16 ± 0.01	0.03 ± 0.01	0.02 ± 0.01	0.02 ± 0.00
Glycine	0.82 ± 0.02	1.34 ± 0.12	1.06 ± 0.02	0.85 ± 0.04	1.20 ± 0.06	1.44 ± 0.03
Threonine	0.15 ± 0.01	0.20 ± 0.01	0.21 ± 0.01	0.17 ± 0.02	0.20 ± 0.01	0.16 ± 0.01
Arginine	0.50 ± 0.01	0.45 ± 0.01	0.55 ± 0.01	0.61 ± 0.02	0.42 ± 0.02	0.40 ± 0.01
Alanine	0.79 ± 0.02	1.08 ± 0.03	0.91 ± 0.01	0.75 ± 0.04	0.96 ± 0.02	0.97 ± 0.03
Tyrosine	0.16 ± 0.01	0.21 ± 0.01	0.21 ± 0.01	0.25 ± 0.01	0.18 ± 0.01	0.15 ± 0.00
Cystine	0.06 ± 0.00	0.08 ± 0.01	0.81 ± 0.02	0.04 ± 0.00	0.07 ± 0.03	0.07 ± 0.03
Valine	0.07 ± 0.01	0.46 ± 0.03	0.36 ± 0.02	0.41 ± 0.01	0.46 ± 0.01	0.39 ± 0.01
Methionine	0.19 ± 0.01	0.22 ± 0.01	0.39 ± 0.03	0.20 ± 0.01	0.18 ± 0.01	0.16 ± 0.00
Phenylalanine	0.20 ± 0.01	0.29 ± 0.02	0.16 ± 0.01	0.35 ± 0.01	0.28 ± 0.01	0.23 ± 0.00
Isoleucine	0.24 ± 0.01	0.41 ± 0.02	0.20 ± 0.01	0.40 ± 0.07	0.39 ± 0.01	0.33 ± 0.01
Leucine	0.33 ± 0.02	1.03 ± 0.03	0.68 ± 0.03	0.97 ± 0.04	0.87 ± 0.02	0.83 ± 0.02
Lysine	0.78 ± 0.00	0.91 ± 0.06	0.65 ± 0.00	1.01 ± 0.10	0.88 ± 0.09	0.87 ± 0.03
Proline	0.51 ± 0.05	0.97 ± 0.08	0.76 ± 0.06	0.59 ± 0.02	0.90 ± 0.12	0.78 ± 0.17
Total AA	6.61 ± 0.10 ^d^	9.87 ± 0.17 ^a^	9.26 ± 0.14 ^b^	8.78 ± 0.13 ^c^	9.07 ± 0.07 ^b^	8.59 ± 0.04 ^c^

^1^ Data are presented as mean ± standard deviation of 9 determinations. ^2^ Data with different small letters (^a–d^) in the same row are significantly different (*p* < 0.05).

**Table 3:** PAH contents (μg/kg) in crispy pork spareribs as affected by oil type and processing condition ^1,2^.

PAH	Raw Pork Spareribs	Marinated Pork Spareribs	Crispy Pork Spareribs			
			Soybean Oil		Palm Oil	
			150 °C/12 min	190 °C/6 min	150 °C/12 min	190 °C/6 min
Acenaphthylene (AcPy)	nd ^3^	nd	0.08 ± 0.00 ^a^	0.07 ± 0.01 ^b^	trace	trace
Acenaphthene (AcP)	nd	nd	trace	trace	nd	nd
Pyrene (Pyr)	3.52 ± 0.25 ^e^	10.66 ± 0.08 ^d^	22.36 ± 1.03 ^a^	20.87 ± 0.24 ^b^	17.89 ± 0.02 ^c^	16.70 ± 0.76 ^c^
Benzo[c]fluorene (BcF)	nd	nd	1.56 ± 0.02 ^b^	1.59 ± 0.01 ^a^	1.56 ± 0.01 ^b^	1.55 ± 0.02 ^b^
Benzo[a]anthracene (BaA)	nd	0.87 ± 0.05 ^d^	3.85 ± 0.01 ^b^	3.87 ± 0.01 ^a^	3.84 ± 0.00 ^bc^	3.84 ± 0.00 ^c^
Benzo[b]fluoranthene (BbF)	nd	1.70 ± 0.26 ^c^	5.27 ± 0.03 ^a^	4.78 ± 0.00 ^b^	4.77 ± 0.01 ^b^	4.77 ± 0.00 ^b^
Benzo[j]fluoranthene (BjF)	nd	trace	10.15 ± 0.00 ^a^	10.15 ± 0.00 ^a^	9.65 ± 0.00 ^b^	9.71 ± 0.10 ^b^
Cyclopenta[cd]pyrene (CcdP)	nd	1.37 ± 0.04 ^d^	32.78 ± 0.70 ^a^	27.92 ± 2.48 ^b^	24.33 ± 0.84 ^c^	22.67 ± 0.09 ^c^
Benzo[ghi]perylene (BghiP)	nd	nd	1.27 ± 0.00 ^a^	1.27 ± 0.00 ^ab^	1.27 ± 0.00 ^b^	1.27 ± 0.00 ^ab^
Dibenzo[a,h]anthracene (DBahA)	nd	nd	1.86 ± 0.0 ^a^	1.85 ± 0.00 ^a^	1.86 ± 0.00 ^a^	1.86 ± 0.01 ^a^
Dienzo[a,e]pyrene (DBaeP)	trace ^4^	trace	0.59 ± 0.06 ^a^	0.55 ± 0.03 ^a^	trace	trace
Dienzo[a,i]pyrene (DBaiP)	trace	trace	5.32 ± 0.10 ^a^	5.37 ± 0.03 ^a^	5.28 ± 0.03 ^a^	5.32 ± 0.07 ^a^
Total	3.52 ± 0.25 ^e^	14.61 ± 0.36 ^d^	85.10 ± 1.58 ^a^	78.21 ± 2.67 ^b^	70.44 ± 0.85 ^c^	67.69 ± 0.83 ^c^

^1^ Data are presented as mean ± standard deviation of 9 determinations. ^2^ Data with different small letters (^a–e^) in the same row are significantly different (*p* < 0.05). ^3^ Not detected. ^4^ LOQ ≥ PAHs levels ≥ LOD.

**Table 4:** PAH precursors contents (μg/kg) in crispy pork spareribs as affected by oil type and frying condition ^1,2^.

PAH Precursors	Raw Pork Spareribs	Marinated Pork Spareribs	Crispy Pork Spareribs			
			Soybean Oil		Palm Oil	
			150 °C/12 min	190 °C/6 min	150 °C/12 min	190 °C/6 min
4,4-dimethyl-2-cyclohexene-1-one	nd ^3^	nd	nd	0.60 ± 0.08 ^a^	0.38 ± 0.04 ^b^	0.50 ± 0.05 ^a^
2-cyclohexene-1-one	1.80 ± 0.10 ^d^	0.76 ± 0.13 ^d^	10.58 ± 1.12 ^a^	5.14 ± 1.35 ^bc^	7.30 ± 1.41 ^b^	4.19 ± 0.85 ^c^
Benzaldehyde	5.21 ± 0.84 ^e^	12.95 ± 0.79 ^d^	275.09 ± 21.29 ^a^	115.53 ± 10.51 ^b^	29.52 ± 2.01 ^c^	21.41 ± 1.46 ^c^
trans,trans-2,4-decadienal	nd	nd	1.74 ± 0.20 ^c^	5.76 ± 1.24 ^a^	4.22 ± 0.49 ^b^	3.95 ± 0.50 ^b^
Total	7.01 ± 0.51 ^e^	13.71 ± 1.83 ^d^	287.42 ± 21.30 ^a^	127.04 ± 12.60 ^b^	41.42 ± 1.53 ^c^	30.05 ± 2.03 ^c^

^1^ Data are presented as mean ± standard deviation of 9 determinations. ^2^ Data with different small letters (^a–e^) in the same row are significantly different (*p* < 0.05). ^3^ not detected.

**Table 5:** A two-factorial analysis of HA and PAH formation in crispy pork spareribs as affected oil type and frying condition.

Factor	DF ^1^	SS ^2^	MS ^3^	F-Value	*p*-Value
HAs					
Frying condition	1	35.90	35.90	28.92	0.0007
Oil type	1	14.14	14.14	11.39	0.0097
Frying condition × Oil type	1	12.12	12.12	9.76	0.0141
PAHs					
Frying condition	1	69.83	69.83	25.30	0.0010
Oil type	1	475.33	475.33	172.24	<0.0001
Frying condition × Oil type	1	12.89	12.89	4.67	0.0627

^1^ Degree of freedom. ^2^ Sum of squares. ^3^ Mean squares.

## Data Availability

Data is contained within the article or Supplementary Materials.

## Supplementary Materials

The following supporting information can be downloaded at: https://www.mdpi.com/article/10.3390/foods13020185/s1, Table S1: Retention time and selected reaction monitoring (SRM) detection parameters of 20 HA standards and internal standard (4,7,8-TriMeIQx) using UPLC-MS/MS; Table S2: Retention time and selected reaction monitoring (SRM) detection parameters of 23 PAH standards and internal standard (Triphenylene) using GC-MS/MS.
