# Supplementary material for: Analysis of Polycyclic Aromatic Hydrocarbons via GC-MS/MS and Heterocyclic Amines via UPLC-MS/MS in Crispy Pork Spareribs for Studying Their Formation during Frying
Source: Foods. 2024 Jan 5;13(2):185. doi: 10.3390/foods13020185 (PMC10814522; doi:10.3390/foods13020185)
Supplement: Supplementary file 1 [file foods-13-00185-s001.zip › foods-2781547-supplementary.pdf]

foods

Supplementary Materials

for

Analysis of Polycyclic Aromatic Hydrocarbons via GC-MS/MS and Heterocyclic Amines via UPLC-MS/MS in Crispy Pork Spareribs for Studying Their Formation during Frying

Yu-Wen Lai[1], Baskaran Stephen Inbaraj[1], Bing-Huei Chen[1,2*]

[1]Department of Food Science, Fu Jen Catholic University, New Taipei City 242062, Taiwan.

[2]Department of Nutrition, China Medical University, Taichung 404328, Taiwan.

[*]Correspondence: Email address: 002622@mail.fju.edu.tw; Tel: +886-2-29053626

Table S1. Retention time and selected reaction monitoring (SRM) detection parameters of 20 HA standards and internal standard (4,7,8-TriMeIQx) using UPLC-MS/MS.

HA compound	Retention time (min)	Precursor ion (m/z)[2]	Quantitation		Confirmation	
			Product ion (m/z)	Collision Energy (V)	Product ion (m/z)	Collision Energy (V)
2-amino-1,6-dimethylimidazo[4,5-b]pyridine (DMIP)	0.50	163.1	148.1	24	105.09	37
2-aminodipyrido-[1,2-a:3′,2′-d]imidazole (Glu-P-2)	0.87	185.1	158.1	25	78.05	37
2-amino-1-methyl-imidazo[4,5-f]quinoline (iso-IQ)	0.70	199.09	184.12	25	156.10	21
2-amino-3-methyl-imidazo[4,5-f]quinoline (IQ)	0.81	199.1	184.12	27	157.00	29
2-amino-3-methyl-imidazo[4,5-f]quinoxaline (IQx)	0.67	200.08	185.12	28	132.14	29
2-amino-3,4-dimethyl-imidazo[4,5-f]quinoline (MeIQ)	1.45	213.11	198.09	27	145.15	29
2-amino-6-methyldipyrido-[1,2-a:3′,2′-d]imidazole (Glu-P-1)	2.15	199.1	92.1	36	172.14	26
2-amino-3,8-dimethyl-imidazo[4,5-f]quinoxaline (8-MeIQx)	1.23	214.1	131.07	41	173.18	24
2-amino-1-methyl-imidazo[4,5-b]quinoline (IQ[4,5-b])	2.10	199.11	183.92	27	115.19	46
2-amino-1,6-dimethyl-furo[3,2-e]imidazo[4,5-b]pyridine (IFP)	2.62	203.08	188.17	25	175.14	22
2-amino-3,7,8-trimethyl-imidazo[4,5-f]quinoxaline (7,8-DiMeIQx)	2.51	228.1	131.13	40	187.15	25
2-amino-3,4,8-trimethyl-imidazo[4,5-f]quinoxaline (4,8-DiMeIQx)	2.68	228.1	213.09	26	187.09	23
9H-pyrido[3,4-b]indole (Norharman)	2.82	169.06	115.09	33	89.05	48
2-amino-3,4,7,8-tetramethyl-imidazo[4,5-f]quinoxaline (4,7,8-TriMeIQx) (IS)[1]	2.82	242.13	145.09	42	201.21	26
1-methyl-9H-pyrido[3,4-b]indole (Harman)	2.83	183.09	115.15	34	89.09	49
2-amino-5-phenylpyridine (Phe-P-1)	3.02	171.09	127.13	30	154.07	21
3-amino-1-methyl-5H-pyrido[4,3-b]indole (Trp-P-2)	2.89	198.11	154.14	30	181.08	24
2-amino-1-methyl-6-phenylimidazo[4,5-b]pyridine (PhIP)	3.02	225.1	210.05	30	140.08	54
3-amino-1,4-dimethyl-5H-pyrido[4,3-b]indole (Trp-P-1)	2.95	212.12	195.14	24	168.09	30
2-amino-9H-pyrido[2,3-b]indole (AαC)	3.13	184.07	140.13	33	167.07	24
2-amino-3-methyl-9H-pyrido[2,3-b]indole (MeAαC)	3.25	198.1	181.14	23	127.13	38

[1]internal standard

[2]mass-to-charge ratio

Table S2. Retention time and selected reaction monitoring (SRM) detection parameters of 23 PAH standards and internal standard (Triphenylene) using GC-MS/MS.

PAH	Retention time (min)	Quantitative ion pair		Qualitative ion pair	
		Precursor ion > Product ion (m/z)[2]	Collision Energy (eV)	Precursor ion > Product ion (m/z)	Collision Energy (eV)
Naphthalene (NaP)	7.90	128 > 102	20	128 > 78	25
Acenaphthylene (AcPy)	14.5	152 > 151	20	152 > 150	35
Acenaphthene (AcP)	15.6	154 > 153	20	153 > 152	20
Fluorene (Flu)	17.6	166 > 165	20	165 > 164	25
Phenanthrene (Phe)	21.8	178 > 176	35	178 > 152	25
Anthracene (Ant)	22.1	178 > 176	35	178 > 152	25
Fluoranthene (FL)	27.9	202 > 200	40	202 > 201	25
Pyrene (Pyr)	29.5	202 > 200	40	202 > 201	25
Benzo[c]fluorene (BcF)	33.6	216 > 215	20	215 > 213	30
Triphenylene (IS)[1]	41.1	228 > 226	30	113 > 112	10
Benzo[a]anthracene (BaA)	42.0	228 > 226	35	113 > 112	15
Chrysene (CHR)	41.6	228 > 226	35	228 > 227	20
5-methylchrysene (MCH)	47.5	242 > 241	40	242 > 239	15
Benzo[b]fluoranthene (BbF)	55.8	252 > 250	40	125 > 124	15
Benzo[j]fluoranthene (BjF)	55.8	252 > 250	40	125 > 124	15
Cyclopenta[c,d]pyrene (CcdP)	58.3	226 > 224	45	113 > 112	15
Benzo[a]pyrene (BaP)	61.2	252 > 250	20	125 > 124	40
Indeno[1,2,3-cd]pyrene (IP)	70.8	276 > 274	45	137 > 136	15
Dibenzo[a,h]anthracene (DBahA)	71.0	278 > 276	40	276 > 274	45
Benzo[ghi]perylene (BghiP)	71.6	276 > 274	45	138 > 137	15
Dibenzo[a,l]pyrene (DBalP)	74.9	302 > 300	40	150 > 149	20
Dibenzo[a,e]pyrene (DBaeP)	75.9	302 > 300	40	150 > 149	20
Dibenzo[a,i]pyrene (DBaiP)	76.5	302 > 300	40	150 > 149	20
Dibenzo[a,h]pyrene (DBahP)	76.8	302 > 300	40	150 > 149	20

[1]internal standard.

[2]mass-to-charge ratio.
